# The Relationship between Cadmium Exposure and Mortality in Postmenopausal Females: A Cohort Study of 2001–2018 NHANES

**DOI:** 10.3390/nu15214604

**Published:** 2023-10-30

**Authors:** Jia-Wei Shi, Deng-Xuan Fan, Ming-Qing Li

**Affiliations:** 1 Shanghai Key Laboratory of Female Reproductive Endocrine Related Diseases, Hospital of Obstetrics and Gynecology, Fudan University, Shanghai 200080, China; sjw951125@163.com; 2 Institute of Obstetrics and Gynecology, Hospital of Obstetrics and Gynecology, Fudan University, Shanghai 200080, China; 3 Department of Gynecology, Hospital of Obstetrics and Gynecology, Fudan University, Shanghai 200080, China

**Keywords:** cadmium, postmenopausal female, mortality, cancer, NHANES

## Abstract

Cadmium is one of the most harmful elements to human health, and the health of postmenopausal females is an important public health issue. However, the correlation between exposure to cadmium and the survival status of postmenopausal women is currently not fully clear. This research intended to explore the correlation between cadmium exposure and mortality among postmenopausal females using a representative sample of the population in the U.S. We drew upon the data of the National Health and Nutrition Examination Survey (2001–2018). Cox’s proportional hazards models and a restricted cubic spline regression (RCS) model were utilized to analyze the correlation between blood and urine cadmium and the mortality of postmenopausal women. Stratified analyses also were conducted to identify the highest risk factor of mortality for the participants. The mean concentration of blood cadmium was 0.59 μg/L, and the mean concentration of urine cadmium was 0.73 μg/g creatinine. Higher cadmium concentrations in blood and urine were significantly related to an increase in all-cause mortality for postmenopausal females after adjustment for multivariate covariates. Furthermore, there was a linear positive correlation between urine cadmium concentrations and cancer mortality, while there was no correlation between blood cadmium and cancer death. The correlation between cadmium concentrations and all-cause mortality is stronger in older, more overweight women with a history of hypertension or smoking. We propose that cadmium remains an important risk factor of all-cause and cancer mortality among postmenopausal females in the U.S. Further decreases in cadmium exposure in the population can promote the health of postmenopausal women and prolong their lifespan.

## 1. Introduction

Menopause refers to the decrease in ovarian function and cessation of menstruation [1,2]. The rapid decline in estrogen secretion after menopause causes a series of symptoms and diseases, including hot flashes and night sweats, psychoneurological symptoms, cardiovascular diseases, etc. [3,4,5]. Most females enter menopause between the ages of 49 and 52 worldwide [6]. With the extension of lifespans, women will spend more time in the postmenopausal phase. Therefore, more attention needs to be paid to the health status of postmenopausal females.

Cadmium is a toxic metal widely distributed in the environment, including certain foods, groundwater, and the ambient air [7,8]. Environmental pollution, especially cadmium pollution in soil, can lead to excessive cadmium content in grains and vegetables, which is a crucial public health issue [9,10]. Cigarette consumption is the main environmental source of exposure to cadmium, while diet is the most important route of exposure to this metal among non-smokers and non-occupationally exposed populations. The widespread heavy metal pollution in soil poses a serious threat to vegetable production and food security [11]. The consumption of contaminated vegetables has become a major pathway for cadmium exposure in the human body [11,12]. Research has reported that the food categories with the highest cadmium intake in Americans are grains and bread, leafy vegetables, potatoes, legumes and nuts, and stem/root vegetables [13]. The metabolism of cadmium is slow in the human body, and most cadmium accumulates in the liver and kidneys, causing harm, especially to the kidneys [14]. It is accepted that cadmium concentration in the urine is a sign of the long-term accumulation of burden in body, while cadmium concentration in the blood is a representative marker of acute cadmium exposure. The adverse effects of daily exposure to low levels of cadmium on health have raised increasing concerns. Cadmium has also been listed as a class I human carcinogen [15].

The relationship between cadmium exposure and health is currently not fully clear. Some studies have suggested that cadmium appeared to be related to increased mortality, even at low-level exposure [16,17]. However, there is currently no research focusing on the correlation between cadmium exposure and mortality in postmenopausal females. Therefore, to better manage the health of postmenopausal women, we explored the correlation of cadmium exposure and mortality in NHANES postmenopausal participants from 2001 to 2018.

## 2. Materials and Methods

### 2.1. Study Population

National Health and Nutrition Examination Survey (NHANES) is a cross-sectional survey performed by National Center for Health Statistics (NCHS) of Centers for Disease Control and Prevention (CDC), which is intended to collect representative nutrition and the health status information of the population. All NHANES protocols were available from the CDC’s National Center for Health Statistics Ethics Review Board. The participants signed informed consent forms.

**Figure 1:**
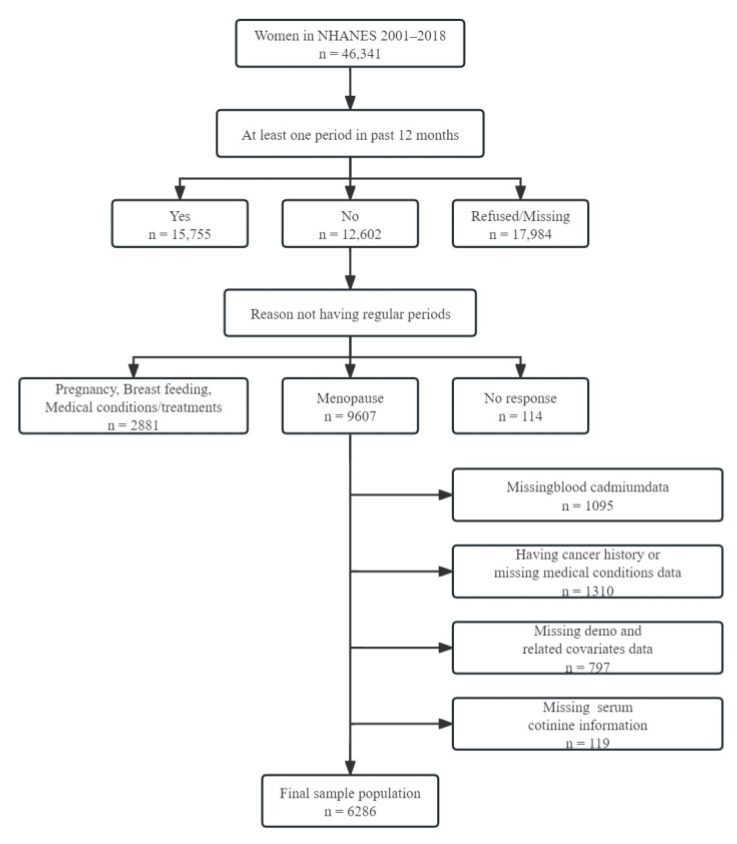
Participant selection flowchart.

For this analysis, 9607 postmenopausal females were enrolled and received interviews and examinations from NHANES from 2001 to 2018. We excluded 1095 participants with no information on blood cadmium measures, 1310 participants with cancer at baseline or with missing data on medical conditions, 797 participants missing base and related covariate data, and 119 participants who lacked serum cotinine information. The final sample population was 6286 participants (Figure 1).

### 2.2. Cadmium Exposure

The blood and urine cadmium were measured at the Division of Environmental Health Laboratory Sciences, National Center for Environmental Health, and Centers for Disease Control and Prevention.

Blood cadmium was measured using an atomic absorption spectrometer using a Zeeman background correction in 2001–2002 and inductively coupled plasma mass spectrometry in 2003–2018. The urine cadmium information was only calculated for about one-third of the randomized subsample of the examined participants (1878 in our study). Mass spectrometry was used to measure urine cadmium. Urine cadmium data were detected in cadmium micrograms per gram of creatinine. All specimen collections, measurements, and analyses followed rigorous procedures, and laboratory details and quality assurance methods are available on the NHANES website (https://www.cdc.gov/nchs/nhanes/, accessed on 25 June 2023).

### 2.3. Determination of Mortality Outcomes

The present study used 2001–2018 NHANES public-use linked mortality files to determine the survival status of the follow-up population, and the survival status of participants was followed up to 31 December 2019. Additionally, ICD-10 was used to ensure disease-specific mortality, and NCHS was used to classify heart diseases (054-068), malignant neoplasms (019-043), and all other causes (010) [18].

### 2.4. Other Variables

Additional covariates were included in our analysis according to previous studies. Data about age, race/ethnicity, education, family income, smoking status, and alcohol intake were collected from self-reported information. The history of diabetes or hypertension was defined based on the laboratory, examination, and questionnaire data.

The variables were divided into the following groups: race/ethnicity (Mexican American, other Hispanic, non-Hispanic White, non-Hispanic Black, other Hispanic, or other race); education levels (less than a high school education, some high school, high school graduate/GED, some college or Associate of Arts (AA) degree, college graduate or more); body mass index (BMI) (underweight (<18.5), normal (18.5 to <25), overweight (25 to <30), obese (BMI 30 or greater)); smoking status (current, former, or never smoker); and alcohol intake (non-drinker, 1 to <5 drinks/month, 5 to <10 drinks/month, or 10+ drinks/month).

Serum specimens were obtained from 2001–2018 NHANES laboratory examinations, for which rigorous procedures were used throughout blood collection and analysis; all the relevant information can be obtained from https://www.cdc.gov/nchs/nhanes/, accessed on 25 June 2023 [19]. We obtained the data on plasma glycohemoglobin (%), glucose (mg/dL), cholesterol (mg/dL), direct HDL-cholesterol (mg/dL), LDL-cholesterol (mg/dL), and triglycerides (mg/dL) from NHANES laboratory examinations.

### 2.5. Statistical Analyses

We converted the blood cadmium and creatinine-corrected urine cadmium data into quartiles. The continuous variables are presented in terms of mean and standard deviation, and the categorical variables are indicated as percentages. Three Cox regression models were created to estimate the relationship between cadmium exposure and mortality: Model 1 (unadjusted); Model 2, which was adjusted for age, race/ethnicity, education level, poverty impact ratio (PIR), BMI, alcohol intake, hypertension, and diabetes; and Model 3, which was adjusted for age, race/ethnicity, education level, PIR, BMI, alcohol intake, hypertension, diabetes, smoking status, and serum cotinine. Schoenfeld residuals was used to detect proportional hazard based on the Cox regression models. The results are shown in Table S1, and *p >* 0.05 indicates that the model is applicable.

Furthermore, a restricted cubic spline regression (RCS) model was constructed to explore the non-linear relationship between blood cadmium, creatinine-corrected urine cadmium concentration, and mortality. Additionally, subgroup analyses were performed based on age (<60 years old or ≥60 years old), race/ethnicity (White or non-White), BMI (< 25.00 or ≥25.00), hypertension, diabetes, and smoking status. R software (version 4.3.1) was used for the above data analysis.

## 3. Results

### 3.1. Characteristics of Participants

The mean concentrations of blood cadmium and urine cadmium were 0.59 μg/L and 0.73 μg/g creatinine. Additionally, the distribution of participants was relatively average based on the quartile of blood cadmium and urine cadmium (Table 1 and Table 2). The blood and urine cadmium concentrations seem positively related to the following factors: age, non-Hispanic White, lower education levels, lower income, less obesity, and current smoking. It is worth noting that blood cadmium and urine cadmium were strongly correlated with smoking status and serum cotinine levels.

In addition, we also explored the indices of glucose and lipid metabolism among participants according to cadmium exposure. Compared to lower blood cadmium, higher blood cadmium levels were correlated with lower concentrations of glucose and with higher HDL at baseline. However, urine cadmium was not related to these indicators (Table 3).

### 3.2. Association of Exposure to Cadmium with Mortality

Next, we investigated the correlation between cadmium concentration in blood and mortality. During the follow-up period of this study, 1416 all-cause deaths occurred, including 381 cardiovascular disease (CVD)-related deaths and 261 cancer deaths (Table 4). Three Cox regression models were constructed to explore the effect of blood cadmium on mortality. After adjusting the relevant covariates, for all-cause mortality, the fully adjusted hazard ratios (HRs) and 95% confidence intervals (CIs) were 1.00 (reference), 0.99 (0.83, 1.18), 1.13 (0.94, 1.36), and 1.39 (1.13, 1.72), according to the classification of quartile of blood cadmium (Model 3). However, there was no relationship between blood cadmium and CVD and cancer mortality.

Due to the complexity of the NHANES sampling, only 1878 individuals in this study had complete urine cadmium information. Therefore, we also calculated the effect of cadmium concentration in the urine on mortality. For all-cause, CVD, and cancer deaths, compared to the lowest quartiles of urine cadmium, the multivariate adjusted HRs and 95% CIs were 1.50 (1.01, 2.23), 0.51 (0.19, 1.39), and 5.70 (1.91, 16.95) in the highest quartiles (Table 5).

### 3.3. Results of Curvilinear Relationship of Cadmium Concentration and Mortality

**Figure 2:**
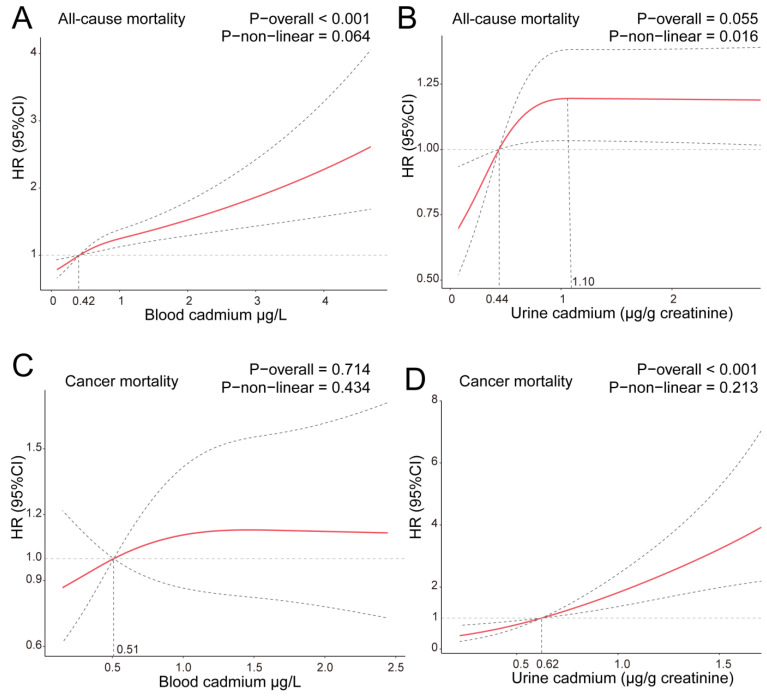
Association between blood cadmium (**A**,**C**) and urine cadmium (**B**,**D**) concentration and all-cause and cancer mortality in postmenopausal females. Adjusted for age, race/ethnicity, education level, PIR, BMI, alcohol intake, hypertension, diabetes, smoking status, and serum cotinine. The solid and dotted lines represent the estimated values and their corresponding 95% CIs, respectively.

To understand whether there is a linear correlation between cadmium concentration in the blood and urine and mortality, we introduced the RCS model. As shown in Figure 2A, there is a linear correlation between blood cadmium and all-cause mortality. However, it seems that there is no linear relationship between urinary cadmium and all-cause death (Figure 2B). Within a certain range, the value of HR is positively associated with urinary cadmium. In other words, the risk of all-cause mortality in postmenopausal women no longer increases when the concentration of urinary cadmium reaches the value of 1.10 μg/g creatinine. In addition, urinary cadmium concentration is positively correlated with cancer mortality in postmenopausal females, while blood cadmium does not exhibit this correlation (Figure 2C,D).

### 3.4. Stratified Analyses

**Figure 3:**
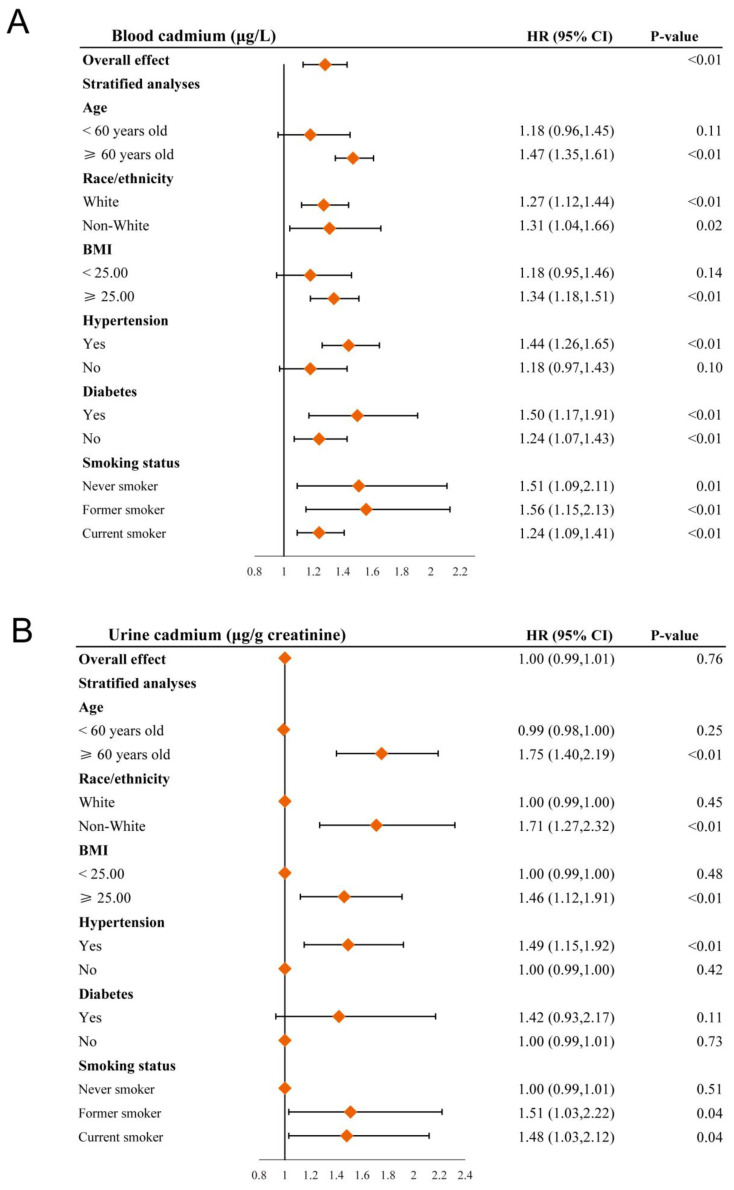
Forest plots of stratified analyses of blood cadmium (**A**), urine cadmium (**B**), and all-cause mortality. Adjusted for age, race/ethnicity, education level, PIR, BMI, alcohol intake, hypertension, diabetes, smoking status, and serum cotinine, except the variable itself.

To further investigate the correlation between cadmium exposure and all-cause mortality, we stratified the data by age, race, BMI, history of hypertension, history of diabetes, and smoking status. In subgroup analysis, cadmium exposure presented a stronger positive correlation of all-cause mortality in older, more overweight women with histories of hypertension (Figure 3A,B). Moreover, as the concentration of cadmium in urine increases, the risk of all-cause death is higher for smokers and non-White populations.

## 4. Discussion

The present research is the first large prospective cohort study aimed at revealing correlations between cadmium exposure and the mortality of postmenopausal females. Our results indicate that cadmium exposure is related to all-cause mortality in the representative population of postmenopausal females after adjustment to related covariates. In addition, compared with the lowest group of urinary cadmium, there is a positive correlation between urinary cadmium level and cancer mortality. The present study does not provide support for the speculation that cadmium exposure increases the risk of CVD mortality in postmenopausal women. In addition, older, more overweight women with a history of hypertension or smoking seem to present a stronger correlation with cadmium concentration in the blood and urine and all-cause mortality.

Cadmium is mainly absorbed through the respiratory and digestive tracts. The cadmium biological half-life in the body is up to 15–30 years, making it the most easily accumulated poison in the body [17]. Cadmium in urine is considered a marker of long-term accumulated burden on the body, while blood cadmium more relies on daily fluctuations in exposure; thus, blood cadmium is defined as a marker of acute exposure [14].

Currently, most studies focus on investigating the mortality rate of individuals exposed to relatively high cadmium levels, while there is limited research on cadmium exposure and mortality in daily life [20,21]. A study on U.S. adults indicated that cadmium has a strong relationship with all-cause and CVD mortality [17]. Additionally, studies on Belgium and Japan populations all suggest that urine cadmium at baseline is related to all-cause mortality [20,21]. However, another report proposed that urinary cadmium is associated with mortality in males but not in females [22].

Menopause refers to the decline in ovarian function and the cessation of menstruation, which is a physiological process that women must undergo. Due to decreasing hormone levels, women become more vulnerable, as low estrogen levels are more likely to cause the occurrence and development of osteoporosis, cardiovascular diseases, and other related diseases among postmenopausal females. Recent studies have shown that metal accumulation in the body can affect the health status of postmenopausal women, but there have been no studies reporting the relationship between cadmium exposure and postmenopausal female mortality rates [23,24,25].

Our results found that postmenopausal women with high concentrations in the blood and urine had a higher risk of all-cause mortality, although the specific physiological mechanisms and related signaling pathways are not yet fully understood. Osteoporosis is not only a health problem that troubles postmenopausal women but also one of the main causes of death in postmenopausal women [26,27]. The existing research suggests that environmental cadmium exposure promotes the occurrence and development of osteoporosis [28,29,30]. Additionally, a systematic review and meta-analysis has indicated that cadmium exposure may be a hazard factor for osteoporosis among postmenopausal females, even at low levels [24]. Based on the above results, osteoporosis caused by cadmium exposure may be one of the reasons for the increased risk of all-cause death in postmenopausal women. Unfortunately, this study lacks complete information on osteoporosis, making it impossible to determine whether cadmium exposure is a decisive factor in postmenopausal women’s osteoporosis leading to death. This will be the focus of our future research work.

Previous studies have suggested that even low-level cadmium exposure remains a determining factor for all-cause and CVD mortality [17]. However, our results did not find a correlation between blood and urine cadmium levels and CVD death in postmenopausal women. The reasons for the above differences may be partly due to differences in the target population, and more importantly, cadmium may be considered a potent metalloestrogen [31,32,33]. Premenopausal women have a low probability of cardiovascular disease due to the protective effect of estrogen with regard to cardiovascular disease. However, during menopause, the endogenous secretion of estrogen decreases, which leads to an increased risk for and incidence rate of CVD [34,35]. Estrogen exerts its cardioprotective effect by reducing oxidative stress and fibrosis and increasing angiogenesis and vasodilation [35,36,37,38]. Additionally, cadmium can increase cell proliferation ability through an estrogen-dependent manner in vitro [31]. Therefore, we speculate that the part of reason why cadmium exposure is not associated with CVD mortality in postmenopausal females may be due to its potential estrogen-like characteristics. Unfortunately, the information regarding estrogen in the NHANES database from 2001 to 2018 is not complete, and our study cannot determine whether sex hormones are involved in the impact of cadmium on the survival status of postmenopausal women. This requires more epidemiological and basic medical research in the future.

In addition, our study shows a positive correlation between urinary cadmium concentration and cancer death among postmenopausal females, which is consistent with previous research findings. Cadmium is a recognized carcinogen for humans and animals. Numerous epidemiological studies have shown that occupational and environmental exposure to cadmium is correlated with the occurrence and progression of various types of cancer [39,40,41]. The main carcinogenic mechanisms induced by cadmium include oxidative stress, epigenetic changes, DNA damage, cell apoptosis, and gene expression changes [39,41,42,43]. This may be a potential mechanism by which cadmium increases the risk of cancer-related death in postmenopausal women. It is worth noting that our study shows that cancer mortality is associated with urinary cadmium but not with blood cadmium. Part of the explanation for the above results may be due to the insufficient sample size, and more importantly, it may also be due to the bioaccumulation of heavy metals, which increases the risk of cancer-related deaths that require long-term chronic cadmium accumulation [44,45].

Identifying sub-populations with a higher risk of all-cause death in postmenopausal women has more significant public health implications. Our study shows that the relationship between exposure to cadmium and all-cause mortality is more pronounced in the elderly, which may be related to the slower metabolism of heavy metals in the elderly. One study has also suggested that the accumulation of cadmium in the body caused by smoking mediates an increased risk of all-cause death [46,47]. Consistently with the above results, our study also found that cadmium concentration is an important determining factor in all-cause mortality among smokers, especially the concentration of cadmium in urine. Therefore, quitting smoking and reducing passive smoking may help reduce cadmium intake and accumulation, improving the health status of postmenopausal women.

The present study has some strengths. Previous research has also reported the correlation between cadmium exposure and population mortality, but for the first time, this study focused on the target group of postmenopausal females. We used NHANES data based on the nationally representative population of the U.S., which had a relatively large sample size and thus enhanced the credibility of results. In addition, the measurement of cadmium concentration in the body strictly followed the standard methods to ensure the accuracy of the data. Finally, the relationship between cadmium concentration and mortality was adjusted for confounding factors, including socioeconomic status, lifestyle, and health status, to ensure the validity of the conclusion. However, our study also has a few limitations. First, the cause and impact are indeterminable due to it being an observational study. Second, there was no long-term observation of cadmium content in the body because the present study measured cadmium concentration at only one time point, which may underestimate the correlation. Third, only one-third of the participants had urine cadmium information, which may lead to small sample sizes and result in errors in the results. Last, our study did not use hormone replacement therapy as an exclusion criterion; thus, we cannot exclude the confounding effects of sex hormone therapy.

## 5. Conclusions

During the past few decades, exposure to cadmium has declined substantially in the United States, but the impact of cadmium on health still deserves more attention [48]. Our study indicates that, compared to low levels of cadmium exposure, high concentrations of cadmium are linked to higher risks of all-cause and cancer mortality in postmenopausal females. In addition, high cadmium concentrations are linked to a higher risk of all-cause death in older, more overweight women with a history of hypertension or smoking. These findings support focusing on cadmium levels in postmenopausal females to help improve their health status.

## Figures and Tables

**Table 1:** Baseline characteristics of participants of postmenopausal women according to blood cadmium in NHANES 2003–2018 data.

	Blood Cadmium (μg/L)					
Characteristics	Total	Quartile 1 <0.29	Quartile 2 0.29–0.41	Quartile 3 0.41–0.66	Quartile 4 >0.66	*p* Value
Participants, *n*	6286 (100.00)	1609 (25.60)	1557 (24.77)	1570 (24.98)	1550 (24.66)	
Age (years)	61.00 ± 10.98	59.00 ± 10.06	61.00 ± 10.69	63.00 ± 11.47	60.00 ± 11.47	<0.01
Race						<0.01
Mexican American	979 (15.57)	298 (18.52)	273 (17.53)	238 (15.16)	170 (10.97)	
Non-Hispanic White	3038 (48.33)	708 (44.00)	768 (49.33)	766 (48.79)	796 (51.35)	
Non-Hispanic Black	1323 (21.05)	366 (22.75)	328 (21.07)	304 (19.36)	325 (20.97)	
Other Hispanic	513 (8.16)	190 (11.81)	121 (7.77)	120 (7.64)	82 (5.29)	
Other race	433 (6.89)	47 (2.92)	67 (4.30)	142 (9.04)	177 (11.42)	
Education						<0.01
Less than 9th grade	923 (14.68)	261 (16.22)	243 (15.61)	231 (14.71)	188 (12.13)	
9–11th grade	944 (15.02)	176 (10.94)	212 (13.62)	220 (14.01)	336 (21.68)	
High school graduate/GED	1625 (25.85)	405 (25.17)	380 (24.41)	410 (26.11)	430 (27.74)	
Some college or AA	1671 (26.58)	451 (28.03)	415 (26.65)	411 (26.18)	394 (25.42)	
College graduate or above	1116 (17.75)	313 (19.45)	306 (19.65)	297 (18.91)	200 (12.90)	
Not recorded	7 (0.11)	3 (0.19)	1 (0.06)	1 (0.06)	2 (0.13)	
PIR	3.03 ± 1.61	3.56 ± 1.58	3.41 ± 1.60	2.94 ± 1.58	2.29 ± 1.60	<0.01
BMI						<0.01
Underweight (<18.5)	84 (1.34)	9 (0.56)	8 (0.51)	20 (1.27)	47 (3.03)	
Normal (18.5 to <25)	1574 (25.04)	301 (18.71)	343 (22.03)	434 (27.64)	496 (32.00)	
Overweight (25 to <30)	1998 (31.78)	487 (30.27)	505 (32.43)	516 (32.87)	490 (31.61)	
Obese (30 or greater)	2630 (41.84)	812 (50.47)	701 (45.02)	600 (38.22)	517 (33.35)	
Alcohol intake						<0.01
Non-drinker	2708 (43.08)	761 (47.30)	710 (45.60)	673 (42.87)	564 (36.39)	
1 to <5 drinks/month	2310 (36.75)	512 (31.82)	587 (37.70)	562 (35.80)	649 (41.87)	
5 to <10 drinks/month	201 (3.20)	48 (2.98)	57 (3.66)	48 (3.06)	48 (3.10)	
10+ drinks/month	500 (7.95)	111 (6.90)	94 (6.04)	130 (8.28)	165 (10.65)	
Not recorded	567 (9.02)	177 (11.00)	109 (7.00)	157 (10.00)	124 (8.00)	
Smoking status						<0.01
Never smoker	3783 (60.18)	1308 (81.29)	1102 (70.78)	939 (59.81)	434 (28.00)	
Former smoker	1591 (25.31)	285 (17.71)	413 (26.53)	527 (33.57)	366 (23.61)	
Current smoker	908 (14.44)	16 (0.99)	41 (2.63)	103 (6.56)	748 (48.26)	
Not recorded	4 (0.06)	0 (0.00)	1 (0.06)	1 (0.06)	2 (0.13)	
Serum cotinine	0.140	0.030	0.033	0.072	6.909	<0.01
(ng/mL; GM (95% CI))	(0.12, 0.16)	(0.027, 0.033)	(0.033, 0.043)	(0.059, 0.088)	(4.940, 9.664)	
Hypertension	3381 (53.79)	874 (54.32)	828 (53.18)	851 (54.20)	828 (53.42)	0.30
Diabetes	1493 (23.75)	447 (27.78)	394 (25.31)	330 (21.02)	322 (20.77)	<0.01

Continuous variables are described as means ± SD, categorical variables are presented as numbers (percentages). Serum cotinine is reported as geometric mean (GM) and 95% confidence interval (CI).

**Table 2:** Baseline characteristics of participants of postmenopausal women according to the urine cadmium in NHANES 2003–2018 data.

	Urine Cadmium (μg/g Creatinine)					
Characteristics	Total	Quartile 1 <0.28	Quartile 2 0.28–0.44	Quartile 3 0.44–0.72	Quartile 4 >0.72	*p* Value
Participants, *n*	1878 (100.00)	470 (25.03)	469 (24.97)	469 (24.97)	470 (25.03)	
Age (years)	60.00 ± 11.25	57.00 ± 11.01	61.00 ± 11.52	61.00 ± 10.81	62.00 ± 10.83	<0.01
Race						<0.01
Mexican American	300 (15.97)	68 (14.47)	71 (15.14)	92 (19.62)	69 (14.68)	
Non-Hispanic White	940 (50.05)	234 (49.79)	246 (52.45)	227 (48.40)	233 (49.57)	
Non-Hispanic Black	382 (20.34)	117 (24.89)	98 (20.90)	89 (18.98)	78 (16.60)	
Other Hispanic	151 (8.04)	37 (7.87)	35 (7.46)	42 (8.96)	37 (7.87)	
Other race	105 (5.59)	14 (2.98)	19 (4.05)	19 (4.05)	53 (11.28)	
Education						<0.01
Less than 9th grade	285 (15.18)	71 (15.11)	73 (15.57)	76 (16.20)	65 (13.83)	
9–11th grade	274 (14.59)	51 (10.85)	53 (11.30)	74 (15.78)	96 (20.43)	
High school graduate/GED	500 (26.62)	122 (25.96)	131 (27.93)	125 (26.65)	122 (25.96)	
Some college or AA	496 (26.41)	127 (27.02)	120 (25.59)	118 (25.16)	131 (27.87)	
College graduate or above	321 (17.09)	98 (20.85)	91 (19.40)	76 (16.20)	56 (11.91)	
Not recorded	2 (0.11)	1 (0.21)	1 (0.21)	0 (0.00)	0 (0.00)	
PIR	2.96 ± 1.59	3.76 ± 1.60	2.89 ± 1.54	2.75 ± 1.57	2.38 ± 1.55	<0.01
BMI						<0.01
Underweight (<18.5)	21 (1.12)	2 (0.43)	3 (0.64)	4 (0.85)	12 (2.55)	
Normal (18.5 to <25)	483 (25.72)	84 (17.87)	122 (26.01)	115 (24.52)	162 (34.47)	
Overweight (25 to <30)	607 (32.32)	128 (27.23)	153 (32.62)	166 (35.39)	160 (34.04)	
Obese (30 or greater)	767 (40.84)	256 (54.47)	191 (40.72)	184 (39.23)	136 (28.94)	
Alcohol intake						0.7
Non-drinker	888 (47.28)	226 (48.09)	223 (47.55)	226 (48.19)	213 (45.32)	
1 to <5 drinks/month	735 (39.14)	181 (38.51)	177 (37.74)	190 (40.51)	187 (39.79)	
5 to <10 drinks/month	69 (3.67)	22 (4.68)	23 (4.90)	9 (1.92)	15 (3.19)	
10+ drinks/month	184 (9.80)	40 (8.51)	46 (9.81)	43 (9.17)	55 (11.70)	
Not recorded	2 (0.11)	1 (0.21)	0 (0.00)	1 (0.21)	0 (0.00)	
Smoking status						<0.01
Never smoker	1138 (60.60)	370 (78.72)	318 (67.80)	274 (58.42)	176 (37.45)	
Former smoker	477 (25.40)	80 (17.02)	112 (23.88)	131 (27.93)	154 (32.77)	
Current smoker	263 (14.00)	20 (4.26)	39 (8.32)	64 (13.65)	140 (29.79)	
Not recorded	0 (0.00)	0 (0.00)	0 (0.00)	0 (0.00)	0 (0.10)	
Serum cotinine	0.146	0.051	0.068	0.202	0.935	<0.01
(ng/mL; GM (95% CI))	(0.115, 0.185)	(0.040, 0.064)	(0.050, 0.095)	(0.121, 0.337)	(0.516, 1.693)	
Hypertension	1023 (54.47)	258 (54.89)	259 (55.22)	256 (54.58)	250 (53.19)	0.7
Diabetes	444 (23.64)	114 (24.26)	109 (23.24)	109 (23.24)	112 (23.83)	>0.9

Continuous variables are described as means ± SD, categorical variables are presented as numbers (percentages). Serum cotinine is reported as geometric mean (GM) and 95% confidence interval (CI).

**Table 3:** Baseline levels of cardiometabolic markers according to cadmium exposure among participants.

	Blood Cadmium (μg/L)				
	<0.29	0.29–0.41	0.41–0.66	>0.66	*p* Value
Glycohemoglobin (*n* = 6269) (%)	5.82 ± 0.99	5.81 ± 0.97	5.81 ± 0.97	5.76 ± 0.86	0.7
Glucose (*n* = 3096) (mg/dL)	109.75 ± 28.14	109.46 ± 34.02	107.49 ± 27.85	106.81 ± 31.89	<0.01
Cholesterol (*n* = 6266) (mg/dL)	208.67 ± 39.91	212.82 ± 40.82	212.22 ± 42.09	211.22 ± 41.58	0.11
LDL (*n* = 3020) (mg/dL)	119.74 ± 35.45	122.17 ± 36.82	124.25 ± 35.66	123.04 ± 37.81	0.14
HDL (*n* = 6265) ((mg/dL))	58.88 ± 16.19	60.95 ± 17.64	61.76 ± 18.39	58.49 ± 17.04	<0.01
Triglycerides (*n* = 3083) (mg/dL)	135.07 ± 113.59	132.61 ± 73.12	126.18 ± 67.48	145.51 ± 104.99	<0.01
	**Urine** **cadmium (μg/g creatinine)**				
	**<0.28**	**0.28–0.44**	**0.44–0.72**	**>0.72**	
Glycohemoglobin (*n* = 1874) (%)	5.72 ± 0.76	5.76 ± 0.97	5.75 ± 0.85	5.77 ± 0.86	0.5
Glucose (*n* = 925) (mg/dL)	104.55 ± 20.28	107.24 ± 30.57	109.25 ± 31.70	104.25 ± 25.98	0.5
Cholesterol (*n* = 1875) (mg/dL)	212.94 ± 42.76	210.77 ± 40.36	214.51 ± 41.10	210.88 ± 43.23	0.8
LDL (*n* = 902) (mg/dL)	115.54 ± 40.01	123.88 ± 32.68	124.90 ± 35.40	122.73 ± 36.56	0.11
HDL (*n* = 1874) ((mg/dL))	60.09 ± 18.16	61.05 ± 16.93	59.94 ± 15.02	60.90 ± 17.46	0.8
Triglycerides (*n* = 924) (mg/dL)	128.46 ± 90.66	132.28 ± 79.95	146.79 ± 85.42	129.31 ± 67.16	0.08

Mean ± SD for continuous variables: *p*-value was calculated via Wilcoxon’s rank–sum test for complex survey samples.

**Table 4:** HRs (95% CIs) for mortality according to cadmium exposure among participants.

	Blood Cadmium (μg/L)				*p* Value
	<0.29	0.29–0.41	0.41–2.19	>2.19	
**All-cause mortality**					
Number of deaths (%)	210 (13.05)	337 (21.64)	398 (25.35)	471 (30.39)	
**Model 1**	1.00	1.30 (1.06, 1.60) 0.01	1.82 (1.50, 2.22) < 0.001	2.19 (1.81, 2.64) <0.001	<0.001
HR (95% CI) *p*-value					
**Model 2**	1.00	1.03 (0.86, 1.23) 0.74	1.24 (1.03, 1.49) 0.02	1.83 (1.50, 2.22) <0.001	<0.001
HR (95% CI) *p*-value					
**Model 3**	1.00	0.99 (0.83, 1.18) 0.94	1.13 (0.94, 1.36) 0.18	1.39 (1.13, 1.72) 0.002	<0.001
HR (95% CI) *p*-value					
**CVD mortality**					
**Number of deaths (%)**	60 (3.73)	88 (5.65)	110 (7.00)	123 (7.94)	
**Model 1**	1.00	0.88 (0.61, 1.26) 0.48	1.03 (0.72, 1.47) 0.86	0.879 (0.56, 1.11) 0.18	0.33
HR (95% CI) *p*-value					
**Model 2**	1.00	0.86 (0.61, 1.21) 0.38	0.94 (0.67, 1.32) 0.72	0.90 (0.63, 1.27) 0.53	0.68
HR (95% CI) *p*-value					
**Model 3**	1.00	0.86 (0.62, 1.21) 0.40	0.95 (0.68, 1.33) 0.76	0.93 (0.65, 1.32) 0.68	0.83
HR (95% CI) *p*-value					
**Cancer mortality**					
**Number of deaths (%)**	38 (2.36)	57 (3.66)	71 (4.52)	95 (6.13)	
**Model 1**	1.00	0.79 (0.47, 1.35) 0.40	0.88 (0.56, 1.41) 0.61	1.10 (0.73, 1.65) 0.64	0.56
HR (95% CI) *p*-value					
**Model 2**	1.00	0.73 (0.44, 1.20) 0.21	0.88 (0.58, 1.34) 0.56	1.19 (0.80, 1.77) 0.40	0.31
HR (95% CI) *p*-value					
**Model 3**	1.00	0.67 (0.41, 1.11) 0.12	0.83 (0.56, 1.23) 0.35	1.11 (0.70, 1.77) 0.65	0.50
HR (95% CI) *p*-value					

Model 1: non-adjusted; Model 2: adjusted for age, race/ethnicity, education level, PIR, BMI, alcohol intake, hypertension, and diabetes; Model 3: adjusted for age, race/ethnicity, education level, PIR, BMI, alcohol intake, hypertension, diabetes, smoking status, and serum cotinine.

**Table 5:** HRs (95% CIs) for mortality according to urine cadmium (μg/g creatinine) among participants.

	Urine Cadmium (μg/g Creatinine)				*p* Trend
	<0.28	0.28–0.44	0.44–0.72	>0.72	
**All-cause mortality**					
Number of deaths (%)	67 (14.26)	107 (22.81)	100 (21.32)	160 (34.04)	
**Model 1**	1.00	1.47 (1.03, 2.09) 0.03	1.48 (0.99, 2.20) 0.05	2.76 (1.96, 3.90) <0.001	<0.001
HR (95% CI) *p*-value					
**Model 2**	1.00	1.06 (0.74, 1.51) 0.76	1.03 (0.69, 1.56) 0.88	1.69 (1.18, 2.43) 0.004	0.01
HR (95% CI) *p*-value					
**Model 3**	1.00	1.04 (0.72, 1.50) 0.84	0.97 (0.64, 1.47) 0.89	1.50 (1.01, 2.23) 0.045	0.08
HR (95% CI) *p*-value					
**CVD mortality**					
**Number of deaths (%)**	20 (4.26)	36 (7.68)	22 (4.69)	37 (7.87)	
**Model 1**	1.00	0.76 (0.38, 1.50) 0.42	0.81 (0.40, 1.65) 0.56	0.50 (0.22, 1.13) 0.09	0.10
HR (95% CI) *p*-value					
**Model 2**	1.00	0.76 (0.38, 1.50) 0.43	0.91 (0.43, 1.95) 0.82	0.48 (0.18, 1.26) 0.14	0.17
HR (95% CI) *p*-value					
**Model 3**	1.00	0.80 (0.40, 1.59) 0.53	0.95 (0.45, 2.02) 0.89	0.51 (0.19, 1.39) 0.19	0.24
HR (95% CI) *p*-value					
**Cancer mortality**					
**Number of deaths (%)**	13 (2.77)	16 (3.41)	16 (3.41)	39 (8.30)	
**Model 1**	1.00	1.58 (0.68, 3.66) 0.28	1.61 (0.52, 5.02) 0.41	1.36 (0.62, 2.99) 0.45	0.50
HR (95% CI) *p*-value					
**Model 2**	1.00	3.40 (1.08, 10.71) 0.04	3.12 (0.90, 10.85) 0.07	5.62 (2.04, 15.47) <0.001	0.002
HR (95% CI) *p*-value					
**Model 3**	1.00	3.06 (0.91, 10.35) 0.07	3.30 (1.00, 10.88) 0.0499	5.70 (1.91, 16.95) 0.002	0.002
HR (95% CI) *p*-value					

Model 1: non-adjusted; Model 2: adjusted for age, race/ethnicity, education level, PIR, BMI, alcohol intake, hypertension, and diabetes; Model 3: adjusted for age, race/ethnicity, education level, PIR, BMI, alcohol intake, hypertension, diabetes, smoking status, and serum cotinine.

## Data Availability

All the data are publicly available at https://wwwn.cdc.gov/nchs/nhanes (accessed on 25 June 2023).

## Supplementary Materials

The following supporting information can be downloaded at: https://www.mdpi.com/article/10.3390/nu15214604/s1; Table S1: The *p* value of Schoenfeld residuals.
