# Supplementary material for: The Relationship between Cadmium Exposure and Mortality in Postmenopausal Females: A Cohort Study of 2001–2018 NHANES
Source: Nutrients. 2023 Oct 30;15(21):4604. doi: 10.3390/nu15214604 (PMC10647223; doi:10.3390/nu15214604)
Supplement: Supplementary file 1 [file nutrients-15-04604-s001.zip › nutrients-2632593-supplementary.pdf]

Table S1 The P value of Schoenfeld residuals.

P-value	Blood cadmium and All-cause mortality		Urine cadmium and All-cause mortality		Urine cadmium and Cancer mortality	
	**Blood cadmium** (μg/L)	**GLOBAL**	**Urine cadmium** (μg/g creatinine)	**GLOBAL**	**Urine cadmium** (μg/g creatinine)	**GLOBAL**
Model1	0.25	0.25	0.82	0.82	0.41	0.41
Model2	0.76	0.40	0.77	0.68	0.94	0.59
Model3	0.48	0.054	0.84	0.23	0.98	0.23

Model 1: Non-adjusted;

Model 2: Adjusted for age, race/ethnicity, education level, PIR, BMI, alcohol intake, hypertension and diabetes;

Model 3: Adjusted for age, race/ethnicity, education level, PIR, BMI, alcohol intake, hypertension, diabetes, smoking status and serum cotinine.
